# Early Dopaminergic Dysfunction Induces PRO-VGF Changes in Blood and Brain of Rats with Alpha-Synuclein Overexpression

**DOI:** 10.1007/s11064-025-04586-6

**Published:** 2025-10-30

**Authors:** Elias Manca, Sara Corsi, Silvia Fanni, Barbara Noli, Antonio Luigi Manai, Giuseppina Bassu, Corda Giulia, Maria Antonietta Casu, Roberto Frau, Pardon Marie-Christine, Graziella Cappelletti, Samanta Mazzetti, Manolo Carta, Cristina Cocco

**Affiliations:** 1 Department of Biomedical Sciences, University of Cagliari, 09042 Monserrato, Cagliari, Italy; 2 CNR-Institute of Translational Pharmacology, 09050 Pula, Cagliari, Italy; 3 School of Life Sciences, University of Nottingham, Nottingham, NG7 2UH UK; 4 Department of Biosciences, University of Milan, Milan, 20122 Italy; 5 Movement Disorder Unit, Fondazione IRCCS Istituto Neurologico “Carlo Besta, Milan, Italy; 6 Guy Everett Laboratory, University of Cagliari, Cagliari, Italy

**Keywords:** ProVGF precursor, Early PD, Alpha-synuclein, Dopamine, Blood biomarker

## Abstract

Previous research revealed a reduction in the VGF immunoreactivity within the substantia nigra (SN) of rats with a 60–90% dopaminergic neuron loss and in the plasma of newly diagnosed Parkinson’s disease (PD) patients. Hence, our aim was to explore whether central and peripheral proVGF changes occur during early dopaminergic dysfunction. To investigate this, we employed a rat model mimicking early-stage PD by injecting the SN unilaterally with an adeno-associated virus (AAV) carrying either the human α-synuclein (α-syn; *n* = 19) or green fluorescent protein (GFP; *n* = 18) gene. After conducting motor assessments and sacrificing the animals, brain and blood samples were collected. Tyrosine hydroxylase (TH)-, glutamic acid decarboxylase (GAD)-, and phosphorylated (p)-α-syn antibodies were used for immunohistochemistry (IHC), while an antibody targeting the C-terminus of proVGF was employed for IHC, western blot (WB), and enzyme linked immunosorbent asssay (ELISA). The α-syn overexpression caused a modest (~ 30%) reduction in TH immunoreactivity within the SN—without affecting the striatum—and did so without producing overt motor symptoms, effectively modeling a pre-symptomatic PD stage. This early dopaminergic dysfunction was accompanied by decreased immunostaining for both proVGF C-trerminus and GAD in the SN, but not the striatum. Reduction in plasma proVGF levels were also observed, indicating systemic changes during initial dopaminergic impairment. Analysis using WB confirmed a decrease in a 70 kDa band consistent with proVGF in both SN and plasma. These findings suggest proVGF as a promising early biomarker for PD, opening new avenues for intervention before the onset of clinical symptoms.

**Supplementary Information:**

The online version contains supplementary material available at 10.1007/s11064-025-04586-6.

## Introduction

Parkinson’s disease (PD) is a neurodegenerative disorder characterized by the progressive loss of dopaminergic neurons in the substantia nigra (SN), forming the nigrostriatal pathway. The neurodegeneration is accompanied by the presence of hallmark aggregates known as Lewy bodies, mainly composed of misfolded α-synuclein (α-syn) proteins, appearing decades before the onset of motor symptoms [1, 2]. Despite the strong support from the Food and Drug Administration (FDA) for utilizing the recently developed α-syn seed amplification assay as an early diagnostic tool [3], currently, there is no approved biological marker for early diagnosis [4]. In recent years, the potential of neuroproteins as promising biomarkers in body fluids has been highlighted, with VGF-derived neuropeptides receiving particular attention [5]. Notably, specific peptides derived from the VGF precursor protein (not to be confused with VEGF), such as those named TLQP [6, 7] and NERP [8], have been identified as potential indicators of amyotrophic lateral sclerosis (ALS) [5, 9], while others, including the AQEE sequence, have been reported at reduced levels in psychiatric disorders [10] but elevated in the context of neurodegeneration associated with multiple sclerosis [11]. We have demonstrated that VGF dysregulation in PD patients particularly impacts the C-terminal region of the VGF precursor (or proVGF) [12]. This was evidenced by a significant reduction we found in VGF levels within the plasma of drug-naïve PD patients, as measured by enzyme linked immunosorbent assay (ELISA) using a specific human C-terminal proVGF antibody [12]. Accordingly, other studies performed with mass spectrometry analyses have identified VGF alterations primarily encompassing the C-terminal region, in urine and cerebrospinal fluid of PD patients [13]. Although these investigations, changes in proVGF or proVGF cleaved peptides in human nigrostriatal circuits have not yet been demonstrated. We have also reported that animal models generated through unilateral injections of either 6-hydroxydopamine [12] or fipronil [14] revealed VGF alterations in the SN along with dopaminergic neurodegeneration. In these rats showing 60–90% neurodegeneration, immunostaining using an antibody to the rat C-terminus of the proVGF was significantly reduced in parallel with a decrease in tyrosine hydroxylase (TH) staining [12, 14]. Furthermore, the toxin-based models used in our previous studies primarily replicate advanced stages of dopaminergic degeneration and have been limited to analyses of brain tissue. Consequently, it remains unclear whether VGF alterations also occur in presymptomatic animal models of PD and whether, in these animals, such changes in the SN are reflected in easily accessible peripheral tissues, such as plasma. Moreover, it is still unknown whether VGF alterations associated with dopaminergic dysfunction involve changes in proVGF itself, as this has not yet been demonstrated in the plasma of PD patients or in advanced PD animal models. Thus, the aim of the present study was to investigate potential proVGF alterations in the rat brain and plasma in response to a ealry dopaminergic dysfunction induced by nigral delivery of a viral vector encoding for the human a-syn gene. This approach better recapitulates PD-related features compared to toxin-based models [15, 16].

## Materials and Methods

### Animals

Male Sprague-Dawley rats (275–300 g, Envigo, Italy) used for adeno-associated virus (AAV) vector treatment were housed under a 12-hour light/dark cycle in a temperature- and humidity-controlled environment, with *ad libitum* access to food and water. Animals were euthanized either by decapitation or via transcardial perfusion with saline (0.9% NaCl), followed by ice-cold 4% paraformaldehyde. Brain samples were collected from both decapitated and perfused animals, while blood was exclusively taken from decapitated animals. Brain areas of interest were collected fresh and immediately snap-frozen on dry ice to preserve molecular integrity and stored at − 80 °C until further processing for Western blot (WB) or ELISA analyses. Blood samples were collected into ethylenediaminetetraacetic acid (EDTA)-coated tubes, centrifuged (4000 g, 10 min), and divided into two aliquots: one stored at − 80 °C for ELISA and the other used for immunoprecipitation before storage for WB analysis. Perfused brains were used for antibody-based techniques aimed at in situ detection of proteins and neurotransmitters, including stereological analyses and proximity ligation assays (PLA). Brain Sect. (40 μm thick) encompassing the SN and striatum were obtained using a microtome. The use of AAV-treated animals was carried out in accordance with the European Directive (EU 2010/63) and Italian D.Lgs (2014/26) and approved by the local committee (OPBA) and the Italian Ministry of Health (829/2019 PR). Homozygous knock-in (HdhQ140^140/Q140^) Huntington’s disease (HD) mice expressing the yellow fluorescent protein (YFP; *n* = 3 males and 4 females) and their YFP littermates (Q140 *n* = 3 males and 5 females) were generated as previously described [17] in the University of Nottingham Bio Support Unit. Blood (approximately 200 µl) was drawn by cardiac puncture under terminal anesthesia and collected in a tube treated with ethylenediaminetetraacetic acid (EDTA, 1.78 mg/ml). Plasma was then separated by centrifugation (3000 g, 10 min) at 4 °C; hence, it was stored frozen (−80 °C). All procedures were carried out in accordance with the UK Animals (Scientific Procedures) Act of 1986 under project license 40/3601 and approved by the University of Nottingham Ethical Review Committee.

###  AAV Vector Treatment

The AAV6 vectors expressing either a human wild-type α-syn or the green fluorescent protein transgene (AAV-α-syn or AAV-GFP) under the human synapsin-1 promoter were used in this study [16]. The viral titer of the AAV-α-syn was 1.7 × 10^14 genome copies/mL, with a working dilution of 50%, whilst the one of AAV-GFP was 2.3 × 10^14, used at the working dilution of 5%. Both vectors were diluted with Dulbecco’s phosphate buffer. Rats were randomly allocated into 3 groups: one was left untreated (*n* = 6), while the other two were selected for unilateral injection in SN with either the AAV-α-syn (*n* = 19) or AAV-GFP (*n* = 18). A small group of rats has been used to test the optimal dilution of the viral vectors (*n* = 10) and perfused 8 weeks after AAV injections. Before AAV injections, animals were deeply anesthetized with a mixture (20:1) of fentanyl (Fentanest, Pfizer) plus the α2 selective agonist medetomidine hydrochloride (Domitor, Orion Pharma) with a dose of 5 mL/kg (intraperitoneal) before being placed in the stereotactic frame. Animals were unilaterally infused with either 3 uL of AAV-α-syn or the AAV-GFP with the aid of a glass capillary fitted into a 22-gauge blunt-tip needle of a Hamilton syringe. The viral vectors were delivered into the right SN at the following coordinates: AP −5.3, ML −2.0, DV −7.2 from the dura mater surface, with the tooth bar adjusted at −2.4. Injections were made at a rate of 0.5 mL/min for a total of 6 min, and the capillary was kept in place for an additional 3 min to avoid diffusion in other areas. Anesthesia awakening was promoted by subcutaneous administration of atipamezole (0.31 mg/kg, Antisedan, Orion Pharma). After the surgery, animals were placed in clean cages and monitored daily until they reached a complete recovery.

### Behavioral Testing of AAV-treated Rats

The naïve (*n* = 6), as well as the AAV-treated animals (*n* = 13 and 8; AAV-α-syn and AAV-GFP, respectively) were subjected to behavioral tests to assess whether any motor impairment had occurred over time due to neurodegeneration. Both the motility and the stepping tests were carried out 8 weeks after viral vector delivery. The locomotor activity test was performed in 41 × 41 × 30 cm transparent plastic cages equipped with 2 orthogonal sets of 16 photocells emitting infrared light beams (Omnitech Digiscan Animal Activity Monitor, Columbus, OH, USA). Animals were individually monitored for a total of 40 min/session and scored on the total distance traveled every 10 min. The stepping test evaluated the number of adjusting steps each animal took when held by an operator, allowing only one forelimb to move freely. The unrestrained paw slides over a smooth surface of 90 cm in 5 s in two directions, as previously described [18]. The rats were tested by a treatment-blinded operator in two consecutive trials consisting of both forehand and backhand directions, and the score was expressed as a percentage of left forelimb use. Animals that did not undergo any surgical procedure (naïve) were utilized as additional control to confirm that no toxicity was yielded by the overexpression of the GFP.

### Stereological Analysis in AAV-treated Rats

Stereological analysis was carried out in the SN of the AAV-treated rats using an anti-TH and Carazzi hematoxylin (CH) staining. Microtome-sectioned slices were incubated overnight with a rabbit anti-TH antibody (1:2000, Merck AB152). The day after, a secondary biotinylated anti-rabbit antibody (1:200, Vector BA-1000) was used over 1 h of incubation. Avidin-biotin complex (Vector PK6100) was exploited to amplify the reaction, and 3′3´-diaminobenzidine chromophore was used in developing the color reaction. For the CH staining, the same sections utilized for the TH stereological analysis were used. In brief, after rehydration, sections were incubated with CH, tap water, and aqueous 1% eosin solution, and after washing in water, they were dehydrated in ascending concentrations of ethanol. Then, the sections were cleared with xylene and coverslipped with Entellan. TH- and CH-stained neurons were counted blindly in the SN pars compacta (SNc) on both hemispheres (injected and contralateral sides) of both AAV-α-syn (*n* = 6) and AAV-GFP (*n* = 4) groups of rats. Systematic, uniform, and random sampling was used to choose sections for analysis. We utilized dedicated software (Stereologer, System Planning and Analysis, Inc., Alexandria, VA) linked to a motorized x, y, z motor stage on a BX-60 Olympus light microscope. The SNc region was outlined at low magnification (×2), and sampling of cells was achieved by using automatically randomized sampling and an optical dissector (50 × 50 × 15 μm). Images were sampled with a ×40 objective through a defined depth with a guard zone of 2 μm. The error coefficient ranged from 0.05 to 0.1. The total number of TH- and CH-stained cells was estimated by means of the optical fractionator method, which combines the optical dissector with the fractionator sampling scheme, giving a direct estimation of the number of 3-D neurons unbiased by their shape, size, and orientation in the entire SNc volume [19, 20].

### Immunofluorescence of proVGF, TH, GAD, and p-α-syn in AAV-treated Rats

Immunofluorescence experiments were carried out in AAV-treated rats with our previously validated rabbit anti-C-terminus proVGF antibody (1:1000; [14]) and antibodies to TH (produced in chicken 1:200; Abcam ab76442), glutamic acid decarboxylase-65 (GAD-65; produced in chicken 1:1,000; Abcam ab139958), and glutamic acid decarboxylase-67 (GAD-67; produced in mouse 1:1,000). Furthermore, as the phosphorylated form of α-syn at serine-129 is the main post-translational modification occurring in Lewy bodies, and it is recognized as a marker of α-syn aggregation, we used an antibody to the S129-phosphorylated form of the α-syn (p-α-syn; produced in rabbit 1:500; Abcam ab51253). Antibodies were used alone or in combination. Sections were first incubated for 1 h with 5% normal donkey serum + 0.25% Triton-100, then incubated overnight with the primary antibodies, while Cy3-conjugated donkey anti-rabbit antibody (1:500, Jackson ImmunoResearch), Alexa488-conjugated donkey anti-chicken antibody (1:300, Jackson ImmunoResearch), or Alexa488-conjugated donkey anti-mouse antibody (1:300, Jackson ImmunoResearch) were used to reveal the primary antibody labeling. Finally, slides were covered with phosphate-buffered saline (PBS; pH ~ 7.4) with glycerol (40%). Routine controls included substitution of each antibody, in turn, with PBS; the use of pre-immune or non-immune sera (in the case of proVGF antibody); and the testing of each secondary antibody with their respective non-relevant primary antibodies. SN slides were photographed using BX51 fluorescence microscopes (Olympus, Milan, Italy) equipped with the FujiS3 Pro digital cameras (Fujifilm, Milan, Italy), while images of the rats’ striatum sections were obtained and digitalized using a Zeiss Axio Z1 slide scanner. Images were captured simultaneously for Cy3 and Alexa488 and then were converted to TIFF format. In order to obtain a semi-quantitative determination of the proVGF-, TH-, and GAD-immunofluorescent signal in the SN and striatum, the FIJI image processing package based on ImageJ (NIH) was used. Briefly, animals per group were selected (6 from the AAV-α-syn group and 4 from the AAV-GFP-injected group), and 4 sections per animal were immunostained with the primary antibodies (anti-TH, proVGF C-terminus, and GAD) and all revelaed with Cy3 staining. The RGB TIFF images were first converted to 8-bit grayscale, then the boundaries of the entire SN, or striatum, were manually traced by the user, and, finally, the background fluorescent signal was removed by a manual thresholding process. The means ± SEM of optical density (OD) values for TH, proVGF C-terminus, and GAD-65/67 were then calculated for each animal group and used for statistical analysis.

### The PLA Analysis of proVGF Staining in AAV-treated Rats

The PLA method has been employed to localize proVGF in neuronal terminals, which are too small for conventional immunofluorescence, to provide accurate colocalization profiles [21]. The experiments were conducted using the AAV-α-syn-treated rats (*n* = 3), where the rabbit anti- proVGF C-terminus antibody (1:2000) was mixed with either the mouse anti-α-syn (1:300) 4D6, mouse monoclonal (Abcam), or the mouse anti-glutamic acid decarboxylase (GAD-67; 1:100, AB_966813) antibodies. All antibodies were diluted in PBS containing 30 mL/L of normal donkey serum, 30 mL/L of normal rat serum, and 0.02 g/L NaN₃. Sections of the SN were incubated overnight in a humid chamber with the primary antibodies. To reveal close proximity between two antigens, the Duolink^®^ kit (Sigma-Aldrich) was used. The sections were then incubated for 2 h at 37 °C with anti-rabbit-PLUS (DUO92002-100RXN, Duolink^®^ In Situ PLA^®^ Probe Anti-Rabbit PLUS, 1:5 in PLA diluent) and anti-mouse-MINUS probes (DUO92004-100RXN, Duolink^®^ In Situ PLA^®^ Probe Anti-Mouse MINUS, 1:5 in PLA diluent). Amplification of the signal was achieved through sequential incubations with (i) ligase in Duolink^®^ ligation solution for 1 h at 37 °C and (ii) polymerase in Duolink^®^ amplification reagents, which were subsequently combined with the secondary antibody, donkey anti-mouse conjugated to Alexa Fluor^®^ 568 (Molecular Probes), for 2 h at 37 °C. Finally, samples were counterstained with TO-PRO^®^−3 (Molecular Probes; 1:1000, 10 min) and mounted using Mowiol^®^ + DABCO. The sections were analyzed using a Nikon spinning disk confocal microscope using a 63x objective. In addition, we used a spinning disk super-resolution by optical pixel reassignment (SoRa) technique using the silicon-immersion 100x objective.

### Investigating proVGF Molecular Weight by WB in AAV-treated Rats

The WB was performed using both SN (pooled samples) and striatum (single samples), comparing injected and contralateral sides from AAV-α-syn-treated rats (*n* = 6 each side) and AAV-GFP-treated rats (*n* = 7 each side). Plasma samples were also used and obtained from AAV- α-syn or GFP treated rats (*n* = 13 and 8, respectively) and naïve rats (*n* = 6). Tissue samples (SN and striatum) were added to a 10 ml/g extraction solution (PBS + protease inhibitor cocktail, 5 ul/ml PBS: PO₄ buffer, 0.01–0.05 M, pH 7.2–7.4 + NaCl 0.15 M protease inhibitor cocktail: Sigma P8340) and promptly homogenized with ultraturrax for one minute. Tissues were kept in ice for 10 min, then the samples were boiled for another 10 min and cooled down before being centrifuged at 3,000 rpm for 15 min at 4 °C; the supernatant was stored frozen until use. Plasma samples were immunoprecipitated with the proVGF C-terminus antibody covalently linked to magnetic iron oxide beads. The proVGF C-terminus IgG immunoglobulins were coupled with BioMag Amine particles (Polyscience, Inc., Warrington, PA) provided with primary amine groups, following the manufacturer’s instructions. Plasma samples were incubated with proVGF C-terminus antibody linked to BioMag Amine particles and rotated for two hours at room temperature. The supernatant was magnetically separated, aspirated, and discarded. Particles were rinsed four times with wash buffer (10 mM Tris, 150 mM sodium chloride, 1 mM EDTA, and 0.1% BSA) and incubated in rotation for two hours at room temperature with 0.1 M acetic acid. The supernatant was magnetically separated, aspirated, saved, freeze-dried, and stored frozen at −80 °C for WB analysis. The BCA Protein Assay Kit (Thermo Scientific) was used to measure the protein concentration. Proteins were diluted in SDS reducing loading buffer so as to load 30 ug of each sample, denatured by boiling for 5 min, and centrifuged for 5 min. Proteins were separated by gel electrophoresis using a precast polyacrylamide gradient gel (NuPAGE 4–12% Bis-Tris Mini protein gel, Thermo Fisher Scientific) for 20 min at 200 volts. Internal MW standards (PageRuler Plus prestained protein ladder 10–250 kDa, Thermo Fisher Scientific) were run in parallel. Proteins were transferred onto polyvinylidene difluoride (PVDF) membranes (Amersham Hybond-P, GE Healthcare) for one hour at 20 volts. Then membranes were blocked with 5% bovine serum albumin (BSA) diluted in TBS (50 mM Tris-base, 150 mM sodium chloride) containing 0.1% Tween-20 (TBS-T) for one hour at room temperature and incubated at 4 °C for one night with rabbit anti- C-terminus proVGF antibody (1:3,000). The next day, membranes were rinsed four times with TBS-T and incubated at room temperature for one hour with the secondary antibody (horseradish peroxidase-conjugated donkey anti-rabbit antibody, 1:10,000; Jackson ImmunoResearch). Finally, the antigen-antibody reaction was revealed using Thermo Scientific Pierce Enhanced Chemiluminescence (ECL) WB substrate. The ImageQuant LAS 4000 (GE Healthcare) was used to detect the chemiluminescence. To perform accurate OD analysis, striatum and SN membranes were reprobed with anti-actin goat antibody (1:1,000, Santa Cruz Biotechnology) to normalize the signal of target proteins. When possible, band intensity was analyzed using Image Studio software (LI-COR, Biosciences).

### Competitive ELISA

The rat proVGF C-terminus assay was performed as previously described [22] using plasma of AAV-α-syn-treated rats (*n* = 13) compared to rats of controls, which included AAV-GFP-treated, naïve rats (*n* = 8 and 6, respectively), and the HD mice (vs. *n* = 7 vs. 8: HdhQ140^140/Q140^ and YFP, respectively). The striatum extract samples (from both injected and contralateral sides) were also used and obtained from either AAV-α-syn- rats (*n* = 13) or GFP- rats (*n* = 8). SN samples were not included on the plates as they were entirely used for WB analysis. Multiwell plates (Nunc, Milan, Italy) were coated with the proVGF C-terminus synthetic nonapeptide and treated with PBS (containing 9% normal serum from the secondary antibody donor species, 0.2 mg/ml sodium azide, and 1 mg/ml EDTA) for 2 h. Primary incubation with proVGF C-terminus antibody (1:5000) was carried out in duplicate, including serial standard dilutions in parallel with samples. Secondary antibody (donkey anti-rabbit biotinylated antibody 1:10,000, Jackson, West Grove, PA, USA), streptavidin-horseradish peroxidase conjugate (1:10,000, Biospa, Milan, Italy), and tetramethylbenzidine (TMB X-traKem-En-Tec, Taastrup, Denmark) as substrates were used to reveal the positive labeling. Hence, the reaction was stopped with HCl (1 mol/L), and the optical density was measured at 450 nm using a multilabel plate reader (Chameleon: Hidex, Turku, Finland). Recovery of synthetic peptides added to plasma at extraction was >85%.

### Statistical Analyses

Statistical analyses were carried out using the StatistiXL software. For data obtained with immunofluorescence, ELISA, and WB, the ROUT method and Shapiro-Wilk normality test were employed to identify and remove any outliers and to assess the normal distribution of the data, respectively. The sample variances were measured with the F-test for equality of variance; hence, individual or pooled variances were used for the two-tailed Student’s t-test or one-way ANOVA for multiple comparisons. For animal behavior analyses, the Kruskal-Wallis and two-way ANOVA tests were used.

For colocalization analysis pearson’s correlation coefficient was used.

## Results

### The AAV-α-syn Delivery Recapitulated a PD Pre-symptomatic Stage

**Fig. 1:**
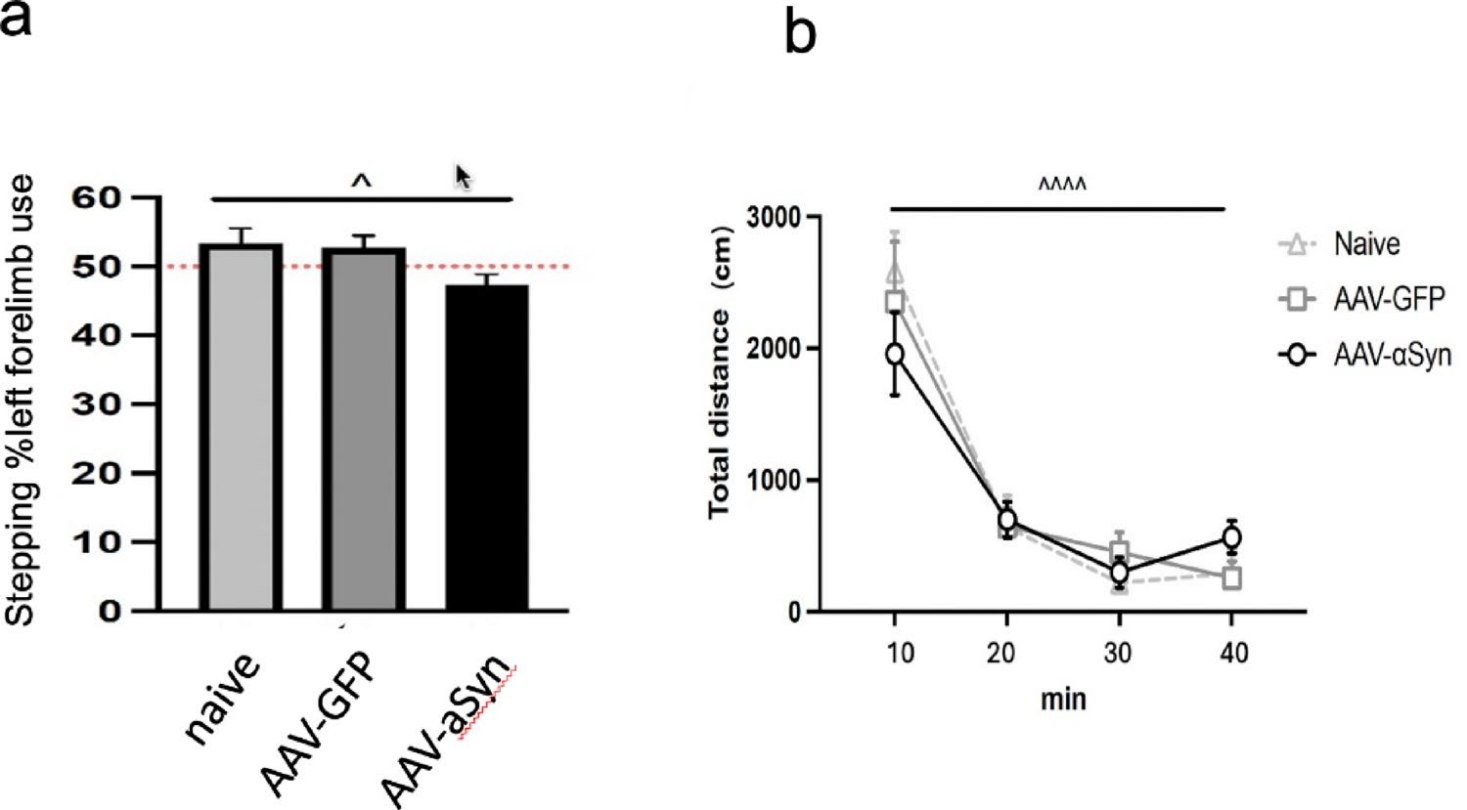
Motor assessment after AAV-α-syn delivery. AAV-mediated α-syn delivery does not produce any motor impairment, suggesting an early stage of neurodegeneration, as highlighted by the percentage of forelimb use in the stepping test (**a** ^, *p* = 0.0363, main effect of treatment, Kruskal-Wallis test) and the total distance traveled in the locomotor activity test (**b** ^^^, *p* < 0.0001, main effect of time, two-way ANOVA RM). AAV-a-syn: adeno-associated virus expressing alpha-synuclein (*n* = 13); AAV-GFP: adeno-associated virus expressing green fluorescent protein (*n* = 8); naïve (*n* = 6) min: minute

Since we sought to determine proVGF-related changes at early stages of PD, we took advantage of a slow-progressing disease-mimicking model based on the overexpression of α-syn in rats. Eight weeks after the transgene delivery, animals were tested for their motor performance at the stepping test and for their general motor activity in an open field. As expected at this early time point, the locomotor activity test revealed no difference in the total distance traveled among the groups (Fig. 1a). The same outcome was obtained when the rats were subjected to the stepping test, which is widely used to highlight unilateral motor impairments in PD models, with no asymmetry detected in the forelimb use of AAV-α-syn animals compared to the naïve or GFP groups (Fig. 1b). These results were in agreement with the limited dopaminergic dysfunction (see below) and support the use of the model employed here as a presymptomatic model of PD.

### The α-syn Overexpression Reproduced early Dopaminergic Dysfunction

**Fig. 2:**
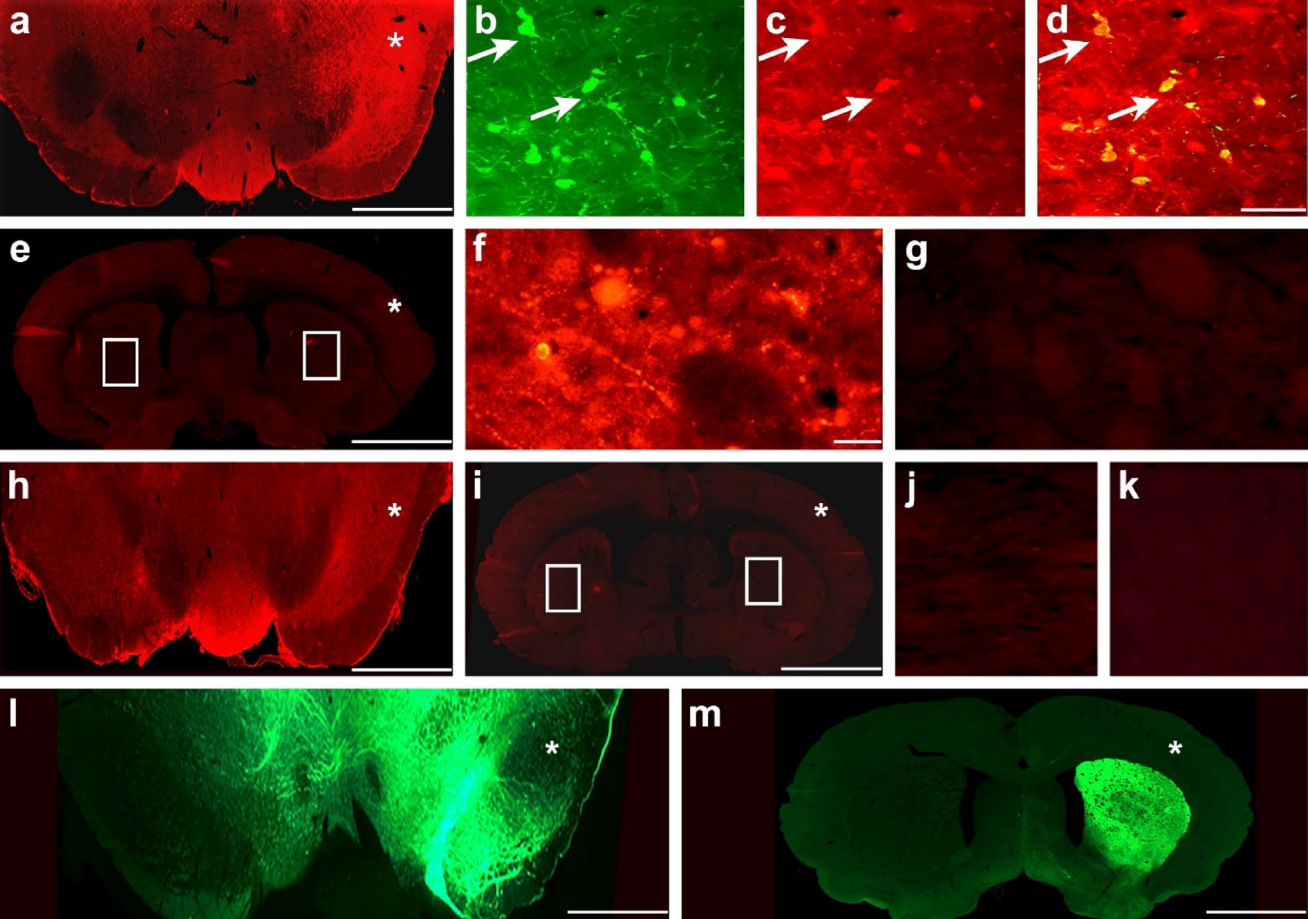
Immunostaining of the p-α-syn antibody in the SN and striatum. In one AAV-p-α-syn-treated rat, the SN revealed that the anti-p-α-syn antibody strongly labeled the injected side, in contrast to the negative staining of the contralateral side (**a**). In the same injected side, dopaminergic cell bodies (**b**): TH antibody, green labeling) exhibited p-α-syn staining aggregates (**c**, anti-p-α-syn antibody, red labeling), as demonstrated by merge double staining (**d**, red and green labeling, anti-p-α-syn and anti-TH antibodies, respectively; colocalization in yellow). In the striatum of the same rat, when observed at low magnification (4x), the p-α-syn antibody appeared negative on both sides (**e**). However, at higher magnification (20x), several aggregates positive for the anti-p-α-syn antibody were visible, predominantly in the dorsal region of the injection site (**f**), while no such aggregates were observed in the contralateral striatum, which remained negative (**g**). In one AAV-GFP-treated rat, no p-α-syn staining was observed on either side of the SN (**h**) nor in the striatum at a high or low magnification (**i**: 4x; j, k: 20x). Instead, the same rat exhibited bright green fluorescence on the injected sides of the SN (**l**) and striatum (**m**), while the contralateral sides did not. Images of the SN and striatum were arranged such that the injected side is marked with an asterisk. Rectangles indicate areas shown at higher magnification. Red labeling: Cy3 fluorochrome; green labeling: Alexa 488 fluorochrome. Scale bars: 2.5 mm (**e**, **i**, **m**);1 mm (**a**, **h**); 0.5 mm (**l**); 50 μm (**b**–**d**, **f**, **g**, **j**, **k**)

**Fig. 3:**
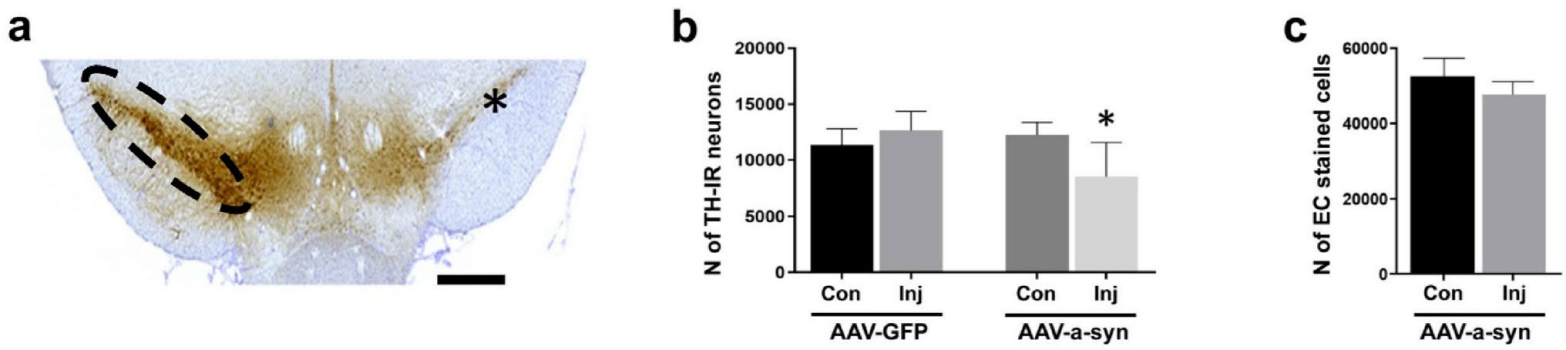
Stereological analysis in SN. The SN section is displayed as an example of the double staining of TH-DAB and CH staining and demonstrates that TH staining was less visible on the injected side compared to the contralateral (**a**). Stereological analysis of the number of TH-positive neurons revealed a significant decrease (**b**, *p* < 0.05) within injected sides of AAV-α-syn compared to the contralateral (*n* = 6 each) and AAV-GFP-treated (*n* = 4 each) rats, while quantification of the CH-positive staining on the same sections showed no statistically significant differences between the two sides of AAV-α-syn-treated rats (**c**, *p* > 0.05). The asterisk in a represents the injected side. N = number; TH-IR: tyrosine hydroxylase immunoreactivity; CH: Carazzi-hematoxylin. AAV-α-syn: adeno-associated virus expressing alpha-synuclein; AAV-GFP: adeno-associated virus expressing green fluorescent protein. Scale bar: 0.5 mm

To confirm the development of α-syn aggregates along the nigrostriatal pathway, we performed immunostaining using an anti-p-α-syn antibody on striatal and nigral sections from rats treated with either AAV-α-syn or AAV-GFP. The SN of the AAV-α-syn-treated rats showed intense labeling on the injected side, in stark contrast to the contralateral hemisphere (Fig. 2a). Dopaminergic neurons in the injected SN developed p-α-syn aggregates, as indicated by the co-localization of TH and p-α-syn immunoreactivity (Fig. 2b–d, white arrows; pearson’s coefficient *r* = 0.762). In the striatum of the same rats, at low magnification, p-α-syn aggregates were scarcely detectable on either side (Fig. 2e). However, at higher magnification, sparse p-α-syn inclusions were observed almost exclusively in the dorsal region of the ipsilateral sides (Fig. 2f), while absent in the contralateral side (Fig. 2g), suggesting a moderate propagation of pathology along the nigrostriatal pathway. The formation of p-α-syn aggregates appeared to be specific to the AAV-α-syn treatment, as using the AAV-GFP-treated rats, no p-α-syn immunoreactivity was detected in either the SN (Fig. 2h) or the striatum (Fig. 2i) at lower or higher magnification (Fig. 2j–k). Instead, these animals exhibited strong GFP fluorescence in the injected regions of both brain areas (Fig. 2l, m). To confirm that our PD animal model revealed limited dopaminergic dysfunction, we performed stereological analysis to quantify TH-positive neurons in combination with (CH)-positive cells (Fig. 3). In AAV-α-syn-treated rats, TH staining was reduced on the injected sides compared to the contralateral sides (Fig. 3a). Stereological analysis of the number of TH-positive neurons revealed a significant decrease (30%; *p* < 0.05) in the injected sides (vs. contralateral sides) of AAV-α-syn-treated rats, but not in AAV-GFP-treated rats (Fig. 3b). Meanwhile, quantification of CH-positive staining on the same sections showed no statistically significant differences between the two sides of AAV-α-syn-treated rats (Fig. 3c, *p* > 0.05). Collectively, these findings demonstrate that AAV-mediated α-syn overexpression recapitulates the α-syn aggregation (characteristic of PD pathology) associated with a moderate TH immunoreactivity reduction in the absence of overt neuronal loss, supporting the presence of a presymptomatic stage of PD, according to the motor behavior analyses.

### Nigral Dopaminergic Dysfunction was Associated with proVGF and GAD Changes

**Fig. 4:**
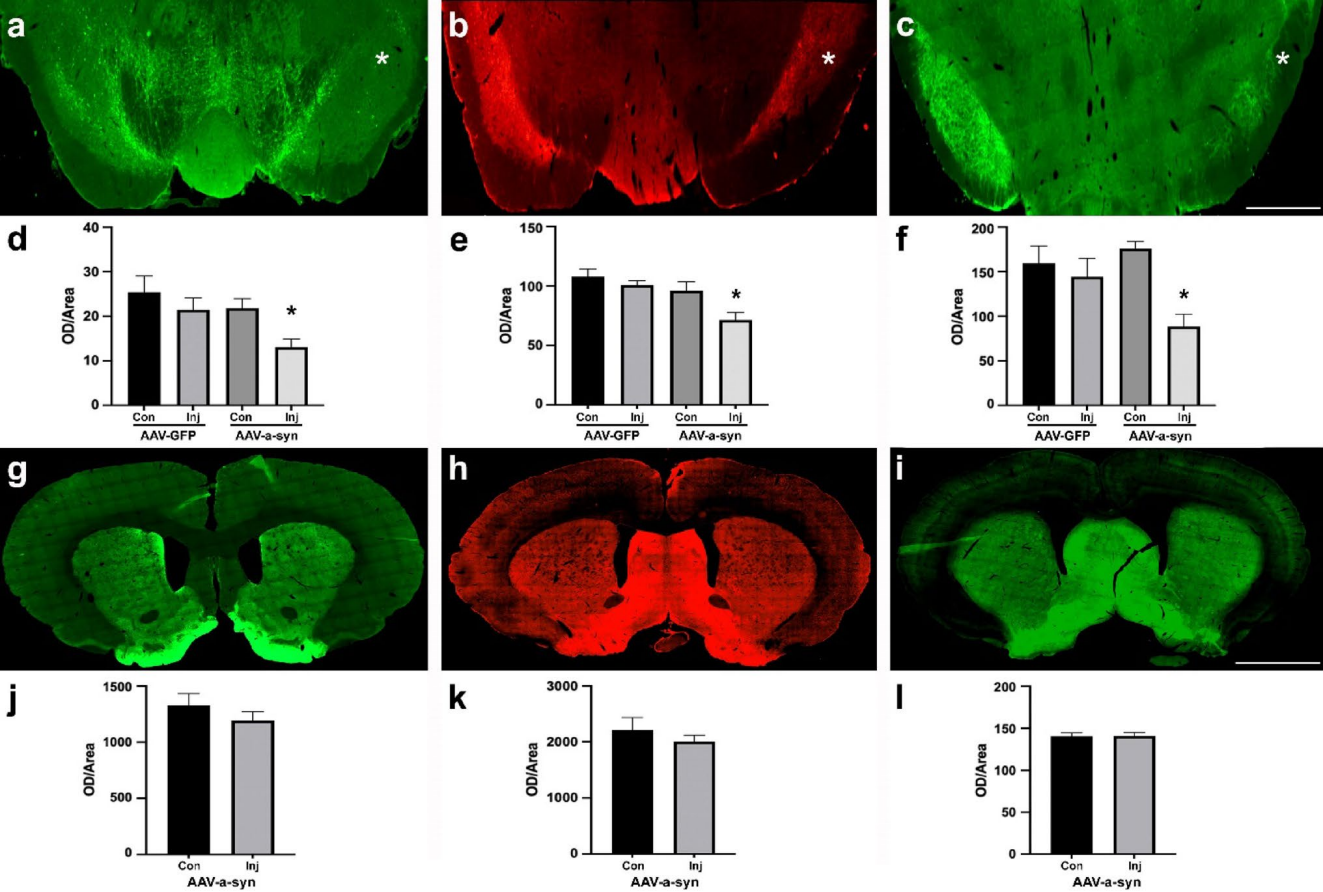
Immunostaining of TH, VGF, and GAD in SN and striatum. In one AAV-α-syn rat, the SN-injected side revealed a reduction (vs. contralateral) in the staining of the tyrosine hydroxylase (**a**, anti-TH antibody, ALEXA 488 green labeling), VGF protein (**b**, Cy3 red labeling; anti-VGF C-terminus antibody), and glutamic acid decarboxylase, (**c**, ALEXA 488 green labeling; anti-GAD-65 antibody). Accordingly, optical density (OD) of the total staining/area (entire SN including compacta and reticulate) showed a reduction in TH and VGF (**d**, **e**; both approximately 30%) as well as GAD immunostaining (f, approximately 50%) within the injected sides of the AAV-α-syn-grat group (*n* = 6; vs. their contralaterals and both sides of *n* = 4 AAV-GFP rats; *p* < 0.05; two-tailed Student’s t-test). In the striatum, a difference was not revealed between the two sides of the same AAV-α-syn rat (injected vs. contralateral) using the antibodies to TH (**g**, ALEXA 488-green labeling), VGF (**h**, Cy3-red labeling), or GAD-67 (**i**, ALEXA 488-green labeling), confirmed by OD analysis of the total striatal area (**j**–**l**, injected vs. contralateral sides) using the same (*n* = 6) AAV-α-syn rat group (two-tailed Student’s t-test). Values are expressed in mean ± SEM; *n* = 4 and 6 for AAV-GFP and AAV-α-syn, respectively. The asterisk represents the injected side. AAV-α-syn: adeno-associated virus expressing alpha-synuclein AAV-GFP: adeno-associated virus expressing green fluorescent protein. *Con* contralateral, *Inj* injected. Scale bars: 1 mm (**a**–**c**); 2.5 mm (**g**–**i**)

Following the validation of the model reflecting a presymptomatic stage of PD, we assessed potential proVGF immunostaining changes in response to the development of pathological p-α-syn aggregates utilizing anti-TH, -proVGF C-terminus, and -GAD antibodies on SN and striatal sections. Consistent with the stereological analysis, we observed a reduction in nigral (entire SN area, including compacta and reticulate) TH immunofluorescence in the AAV-α-syn rats on the injected sides (Fig. 4a). Additionally, on the same sides, we detected a decrease in proVGF (Fig. 4b) and GAD (Fig. 4c) immunoreactivities, suggesting an alteration in GABA and proVGF production alongside the dopaminergic deficits. The reduction in TH immunolabeling occurred primarily around the AAV injection site, which also coincided with the decrease in proVGF labeling (supplemental materials, Fig. 1). The OD analysis applied using the three above antibodies on AAV-α-syn- and AAV-GFP-treated rats confirmed the reduction in TH labeling observed with stereological analysis (30%) and revealed a statistically significant 30% drop in proVGF, along with a greater reduction in GAD-65 staining (approximately 50%) in the injected sides of AAV-α-syn rats (compared to their contralateral sides) (Fig. 4d–f). The changes appeared to be dependent on the α-syn overexpression, as no alterations in TH, GAD, or proVGF immunostaining were observed in the AAV-GFP-treated rats (Fig. 4d–f). The alterations observed in the SN were not mirrored in the striatum, where TH, proVGF, and GAD immunolabeling remained unchanged in the AAV-α-syn rats (Fig. 4g–i), as confirmed by OD analysis (Fig. 4j–l).

### ProVGF Exhibits Colocalization with GABA and α-syn

**Fig. 5:**
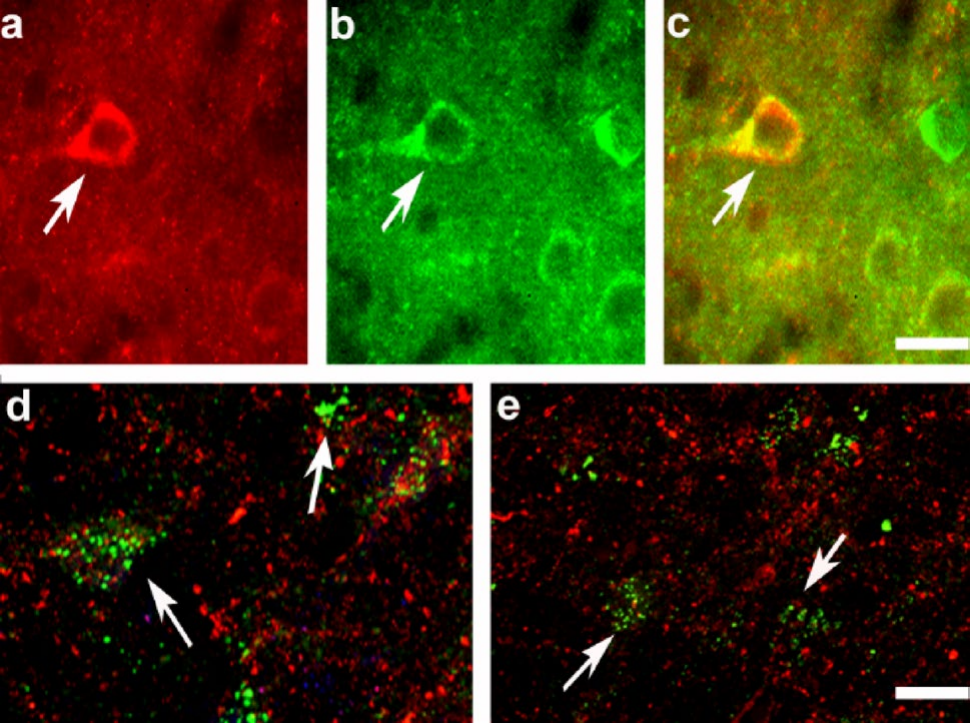
Colocalization analysis in SN and striatum. In the dorsal striatum of the homologous injected side of one AAV-α-syn rat, VGF labeling (**a**; Cy3, red labeling) was found within GAD-67-containing cell bodies (**b**, Alexa 488, green labeling), as shown by merge staining (**c**, GAD-67 plus VGF: yellow labeling). Proximity ligation assay (PLA) using C-terminus of proVGF and GAD-65 antibodies (green labeling) and immunofluorescence for VGF (red labeling) revealed neurons containing both VGF and GAD-65 (**d**). The PLA using anti-VGF and anti-α-syn antibodies and immunofluorescence for VGF showed red labeling (VGF staining alone) and green labeling, indicating neurons containing both VGF and α-syn (**e**). Scale bars: 25 μm, (**a**–**c**) 10 μm (**d**–**e**)

Given our previous finding that VGF C-terminus immunoreactivity was absent in dopaminergic neurons [12], we carried out double immunostaining in the striatum and SN to specifically evaluate the association of proVGF with GAD or α-syn. In the striatum, the most likely site of proVGF production, combined immunolabeling revealed proVGF-positive cell bodies that also expressed GAD (Fig. 5a–c). To more precisely characterize the colocalization profile in the SN, we employed the PLA, as conventional immunofluorescence lacks the spatial resolution needed to reliably detect molecular proximity in small structures such as nigral neuronal terminals, where proVGF is likely secreted from the striatum. In the injected sides of AAV-α-syn-treated rats, PLA revealed the close spatial association of proVGF with GAD within SN neuronal terminals (Fig. 5d), supporting previous observations [12] as well as a close proximity between proVGF and α-syn (Fig. 5e).

### ProVGF is the Specific VGF Isoform Affected in the Brain

**Fig. 6:**
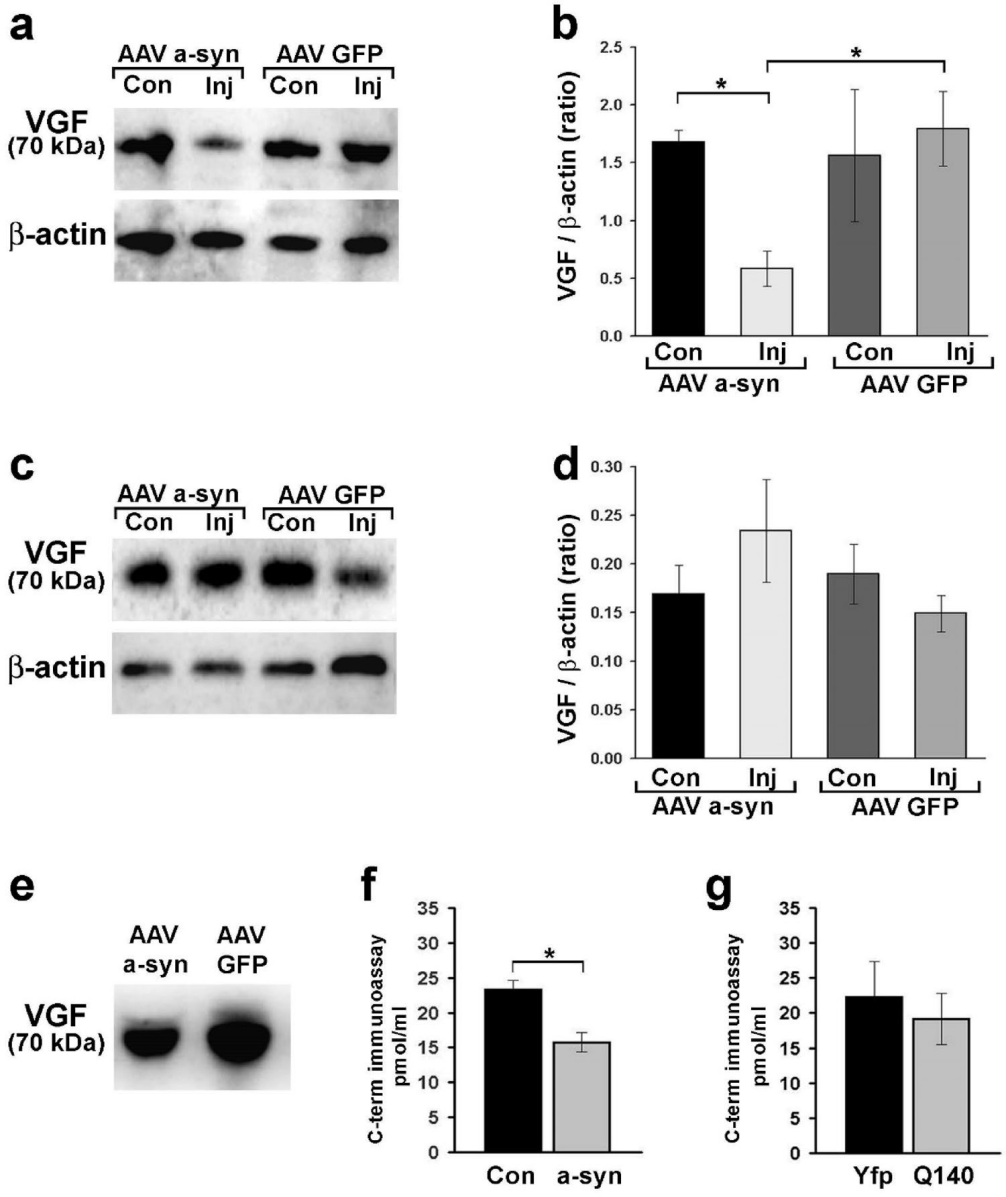
ProVGF changes in the brain and plasma extracts. WB analysis was performed using pooled samples of the SN, comparing the injected and contralateral sides of AAV-α-syn-treated rats (pooled samples from *n* = 6 per side) and AAV-GFP-treated rats (pooled samples from *n* = 7 per side). A band of 70 kDa, which corresponds to proVGF, was less labeled by the VGF antibody using the injection sides of the AAV-α-syn rats (**a**, vs. their controlateral sides and the AAV-GFP sides). Densitometric analysis using the same samples confirmed the 70 kDa band reduction in AAV-α-syn injection sides (**b**; *p* < 0.05). Using striatal samples from one AAV-α-syn-rat and one AAV-GFP- rat (both including injected and contralateral sides) as representative examples, no differences in the proVGF band were observed (**c**), as confirmed by densitometric analysis (**d**) comparing the AAV-α-syn- rat group (*n* = 6 per side) and the AAV-GFP- rat group (*n* = 7 per side). In plasma samples from one AAV-α-syn rat and one AAV-GFP rat taken as representative examples, the band of 70 kDa corresponding to proVGF was visibly reduced using the AAV-α-syn rat (**e**). Such a difference was confirmed with ELISA (**f**) using plasma of the AAV-α-syn-treated rat group (*n* = 13) compared to rats of controls, which included AAV-GFP-rat group (*n* = 8) and the naïve rats (*n* = 6); (*p* = 0.001). Instead, no statistically significant VGF changes were observed between the Q140 (*n* = 3 males and 5 females) and YFP (YFP; *n* = 3 males and 4 females) mice. Values are expressed as mean ± SEM. Abbreviations: Con: contralateral; Inj: injected. AAV-α-syn: adeno-associated virus expressing alpha-synuclein; AAV-GFP: adeno-associated virus expressing green fluorescent protein

To reveal if the VGF changes in the brain were due to the proVGF modification, we performed WB analysis on samples from the SN and striatum. In the SN pooled samples, WB revealed a significant decrease of the 70 kDa band—corresponding to the proVGF protein—on the injected side of AAV-α-syn-treated rats compared to the contralateral side (Fig. 6a), corroborating the immunohistochemistry findings. These results were also ensured by densitometric analysis (Fig. 6b). The reduction in proVGF levels appears to be dependent on α-syn overexpression, as animals overexpressing GFP showed no such decrease (Fig. 6a, b). Moreover, proVGF levels on the contralateral side of AAV-α-syn rats were comparable to those in AAV-GFP controls, indicating that these changes are specific to neuronal dysfunction (Fig. 6b). Regarding WB analysis see also Supplemental materials Fig. 3. In contrast, proVGF levels in the striatum remained unchanged at this presymptomatic stage (Fig. 6c), with no significant differences detected using densitometric analysis between the AAV-α-syn and AAV-GFP groups (Fig. 6d), a result further confirmed by ELISA (Supplemental materials, Fig. 2).

### Peripheral proVGF Alterations Reflect Early PD Brain Dysfunction

Finally, we aimed to investigate whether the proVGF alterations observed in the SN were paralleled by changes in proVGF plasma levels. Each plasma sample from AAV-GFP or AAV-α-syn rats was analyzed using WB following immunoprecipitation with an antibody targeting the C-terminus of the proVGF. A distinct 70 kDa band, corresponding to full-length proVGF, was detected in all samples. However, plasma samples from the AAV-α-syn-injected animals contained lower levels of proVGF compared to the AAV-GFP group (Fig. 6e), similar to what was observed in the SN.

Regarding WB analysis see also Supplemental materials Fig. 3. ELISA using the same plasma samples confirmed these findings, with approximately a 30% reduction in the AAV-α-syn-overexpressing group compared to controls (both AAV-GFP and naïve rats; *p* < 0.05; approximately 30% decrease; Fig. 6f). Finally, to assess whether the decrease in VGF could be specific to PD, we performed a proVGF C-terminus assay in plasma samples from a genetic model of HD and compared them to their controls. No significant difference in proVGF C-terminus levels was observed between the two groups (Fig. 6g), suggesting that the alteration could be specific to PD.

## Discussion

In the present study, we demonstrate for the first time that proVGF alterations can be detected even in the absence of significant dopaminergic cell loss or overt motor deficits. The preclinical model employed here mimics the human PD pathology more accurately than classical toxin-based models, as it replicates a pre-symptomatic stage of the disease with the formation of pathological p-α-syn aggregates characteristic of PD [15, 16]. Furthermore, it allows the investigation of different stages of the disease, thereby capturing, opposite to toxin-based models, an early stage. Interestingly, the modest dopaminergic dysfunction observed in this model was accompanied by proportional alterations in nigral proVGF immunoexpression. Specifically, a significant decrease in TH-positive neurons was observed in the SNc. To determine whether the reduction in TH-positive neurons was due to the inhibited TH protein expression or actual degeneration of dopaminergic neurons, we also performed stereological quantification of CH-stained cells, finding no differences between the injected and contralateral sides. The discrepancy between the reduction in TH-positive neurons and the preservation of CH-positive cells suggests that, at this time point, the decrease in TH expression occurs without overt neuronal loss. Moreover, we did not observe any reduction in the TH immunoreactivity within the striatum. The earliest sign of degeneration appears as swollen and fragmented fibers in the striatum, positive for p-a-Syn aggregates. While quantification of dopamine levels or functional assessment would shed light upon the alterations in striatal dopaminergic terminals, the presence of p-a-syn aggregates, combined with the lack of reduction of TH + fibers, points toward a pre-degenerative state. Consistently, in our previous studies, sections from rats treated with toxins (6-OHDA and fipronil) that exhibited approximately 60–90% dopaminergic neuron loss also showed a comparable 60–90% reduction in proVGF C-terminus immunoreactivity, using the same VGF antibody [12, 14]. Interestingly, the PLA technique confirmed a close spatial association between proVGF and α-syn or GAD within SN neuronal terminals, suggesting the presence of α-syn in GABAergic nigral neurons, given that proVGF immunoreactivity is not observed in dopaminergic neurons [12]. These findings raise intriguing questions, and further targeted investigations are warranted, to determine whether GABAergic neurons can harbor p-α-syn aggregates. The WB analysis we performed has demonstrated for the first time that the proVGF isoform is present not only in the SN but also in the plasma thanks to our immunoprecipitation strategy. The concurrent reduction of the same proVGF form in both SN and plasma suggests a potential contribution of nigral proVGF release to circulating levels, although further studies are required to establish a direct causal link. Notably, proVGF levels in plasma were reduced to a degree comparable to that observed in the SN, despite the viral vector being unilaterally injected. This finding may indicate a broader systemic effect or inter-hemispheric communication. Additionally, WB analysis confirmed the presence of proVGF in the striatum; however, no significant changes in its levels were detected in this region. Given that p-α-syn aggregates are known to induce functional synaptic deficits [23], and considering that proVGF is stored in dense core vesicles and released into the synaptic space [24, 25], the observed SN alterations may reflect synaptic dysfunction associated with early PD-like pathology. In contrast to the SN, the striatum showed a moderate level of p-α-syn immunostaining, suggesting that the p-α-syn was not expressed at a sufficient level to cause proVGF damage, according to the mild dopaminergic dysfunction and the absence of behavioral deficits. Another important point to consider is that the alterations detected using the same proVGF C-terminus antibody appear to be specifically associated with dopaminergic dysfunction within the nigrostriatal pathway. As previously reported, proVGF C-terminus plasma levels detected with the same antibody used in this study did not change in the phencyclidine rat models of schizophrenia [22], nor in the plasma of patients with early-stage ALS or in animal models of early motor neuron degeneration, conditions in which other VGF-derived peptides were altered [5, 26, 27]. Further supporting this specificity, here we also demonstrated that proVGF C-terminus levels remained unchanged in an animal model of HD. In conclusion, we have shown that, in the context of a early dopaminergic dysfunction induced by AAV-mediated overexpression of α-syn, we have found a significant reduction in the SN of the proVGF isoform expressed within GABAergic neurons. Most importantly, a potential link between brain and blood compartments is suggested since the central reduction of the same proVGF isoform is mirrored by a similar decrease in its peripheral levels in our PD animal model but not in HD animals. If confirmed in clinical settings, these early alterations in peripheral proVGF levels could provide a valuable tool for identifying the pathological process long before the onset of motor symptoms, offering a window for earlier intervention.

## Supplementary Information

Below is the link to the electronic supplementary material.

Supplementary file1 (JPG 1220 KB)—VGF and TH staining through the SN sections. Analysis of the entire SN showed that the reduction in TH labeling especially occurred near the AAV-α-syn-injection site, which also coincided with the decrease in VGF labeling. VGF and TH labeling were revealed with Cy3 (red labeling) and ALEXA488 (green labeling), respectively. Scale bar: 400 μm. c, h, and m are taken from Paxinos and Watson (1998)

Supplementary file1 (JPG 1220 KB)—VGF-ELISA levels in striatum. Striatum samples were obtained from injected and contralateral sides of AAV-α-syn-treated rats (n = 6 each side) as well as GFP-treated rats (n = 7 each side). No changes were revealed using the AAV-α-syn-treated rats (p >0.05; 43 ± 2 and 43.87 ± 2 injected vs. contralateral, respectively) nor between the AAV-GFP groups (p >0.05). Con: contralateral; Inj: injected. AAV-α-syn: adeno-associated virus expressing alpha-synuclein; AAV-GFP: adeno-associated virus expressing green fluorescent protein. Data are presented as means ± SEM

Supplementary file1 (JPG 1220 KB)—Original membrane images of WB using brain samples. Original WB membrane images of the substantia nigra (SN) and striatum used to generate Fig. 6 are shown, including the corresponding β-actin. AAV-α-syn: adeno-associated virus expressing alpha-synuclein; AAV-GFP: adeno-associated virus expressing green fluorescent protein; inj: injected side; con: contralateral side.‘I’ and ‘II’ refer to two pooled SN samples, while ‘1’ and ‘2’ represent individual samples

Supplementary file1 (JPG 1220 KB)—Original membrane images of WB using plasma samples. Original WB membrane images of the plasma samples used to generate Fig. 6 is shown. Plasma samples were analyzed from 4 AAV-GFP-treated and 4 AAV-α-syn-treated rats. AAV-GFP: adeno-associated virus expressing green fluorescent protein; inj: injected side; con: contralateral side

## Data Availability

No datasets were generated or analysed during the current study.
