# Supplementary figures and images for: Early Dopaminergic Dysfunction Induces PRO-VGF Changes in Blood and Brain of Rats with Alpha-Synuclein Overexpression
Source: Neurochem Res. 2025 Oct 30;50(6):344. doi: 10.1007/s11064-025-04586-6 (PMC12575455; doi:10.1007/s11064-025-04586-6)

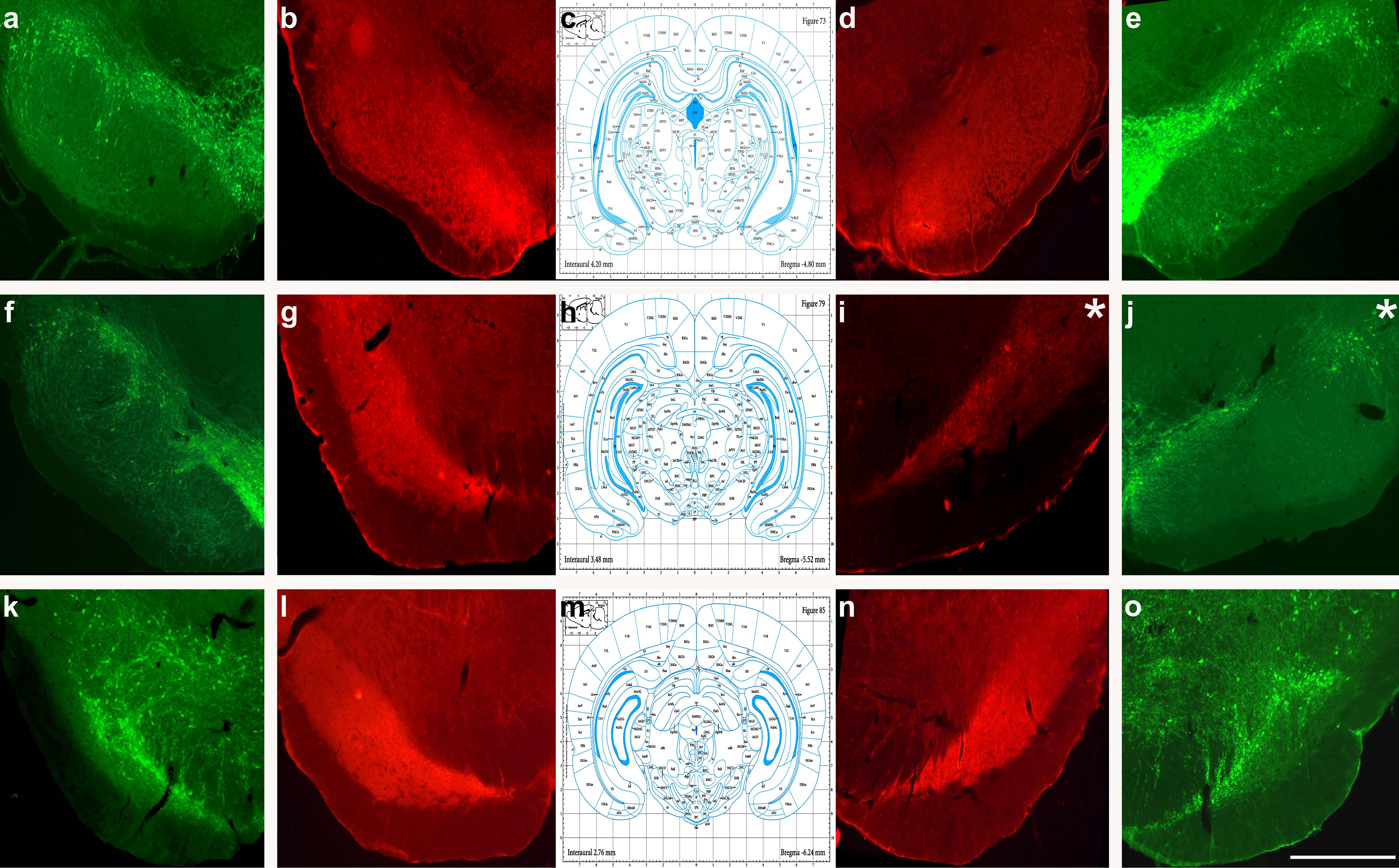

Supplement: Supplementary file 1 — Supplementary file1 (JPG 1220 KB)—VGF and TH staining through the SN sections. Analysis of the entire SN showed that the reduction in TH labeling especially occurred near the AAV-α-syn-injection site, which also coincided with the decrease in VGF labeling. VGF and TH labeling were revealed with Cy3 (red labeling) and ALEXA488 (green labeling), respectively. Scale bar: 400 μm. c, h, and m are taken from Paxinos and Watson (1998) [file 11064_2025_4586_MOESM1_ESM.jpg]

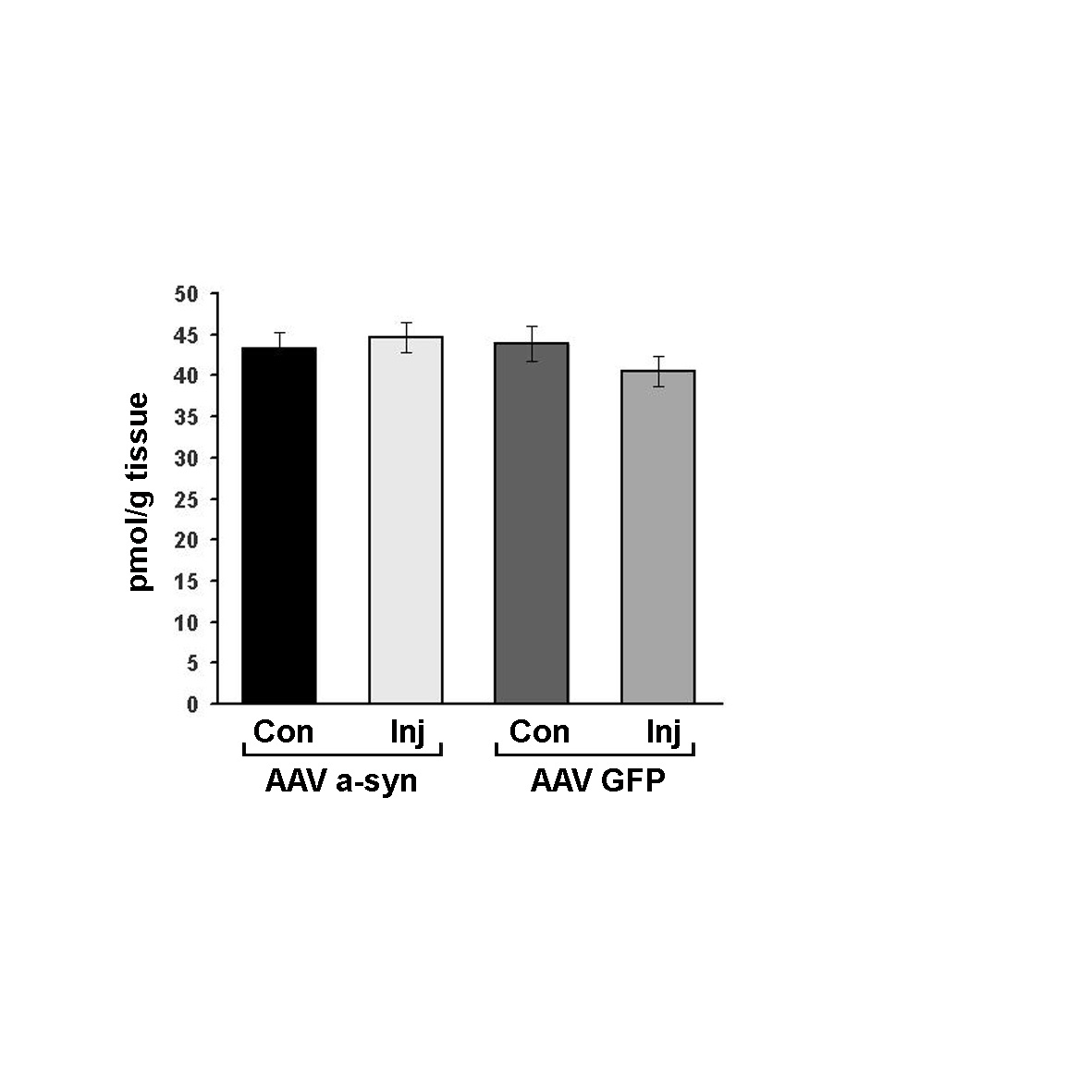

Supplement: Supplementary file 2 — Supplementary file1 (JPG 1220 KB)—VGF-ELISA levels in striatum. Striatum samples were obtained from injected and contralateral sides of AAV-α-syn-treated rats (n = 6 each side) as well as GFP-treated rats (n = 7 each side). No changes were revealed using the AAV-α-syn-treated rats (p >0.05; 43 ± 2 and 43.87 ± 2 injected vs. contralateral, respectively) nor between the AAV-GFP groups (p >0.05). Con: contralateral; Inj: injected. AAV-α-syn: adeno-associated virus expressing alpha-synuclein; AAV-GFP: adeno-associated virus expressing green fluorescent protein. Data are presented as means ± SEM [file 11064_2025_4586_MOESM2_ESM.jpg]

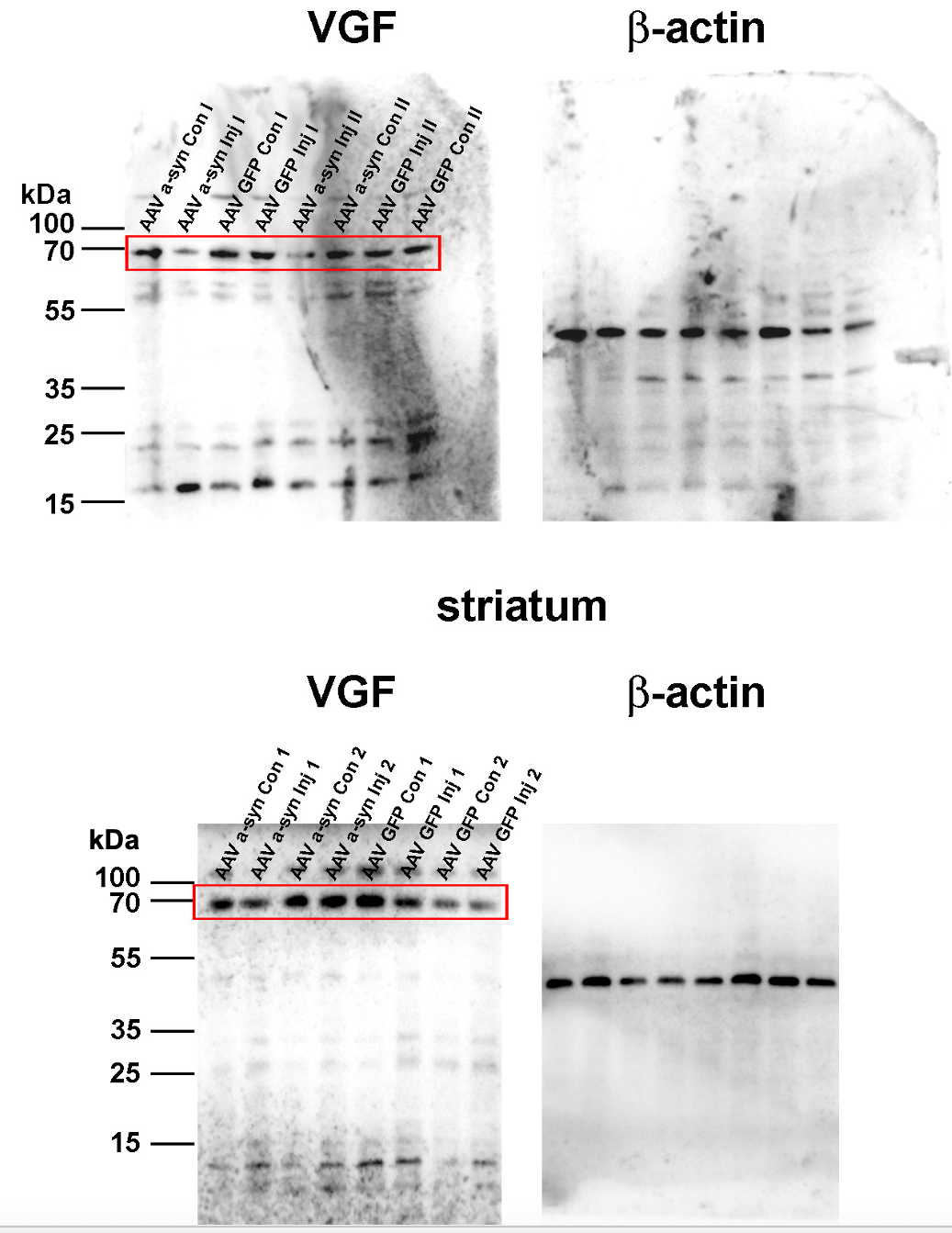

Supplement: Supplementary file 3 — Supplementary file1 (JPG 1220 KB)—Original membrane images of WB using brain samples. Original WB membrane images of the substantia nigra (SN) and striatum used to generate Fig. 6 are shown, including the corresponding β-actin. AAV-α-syn: adeno-associated virus expressing alpha-synuclein; AAV-GFP: adeno-associated virus expressing green fluorescent protein; inj: injected side; con: contralateral side.‘I’ and ‘II’ refer to two pooled SN samples, while ‘1’ and ‘2’ represent individual samples [file 11064_2025_4586_MOESM3_ESM.png]

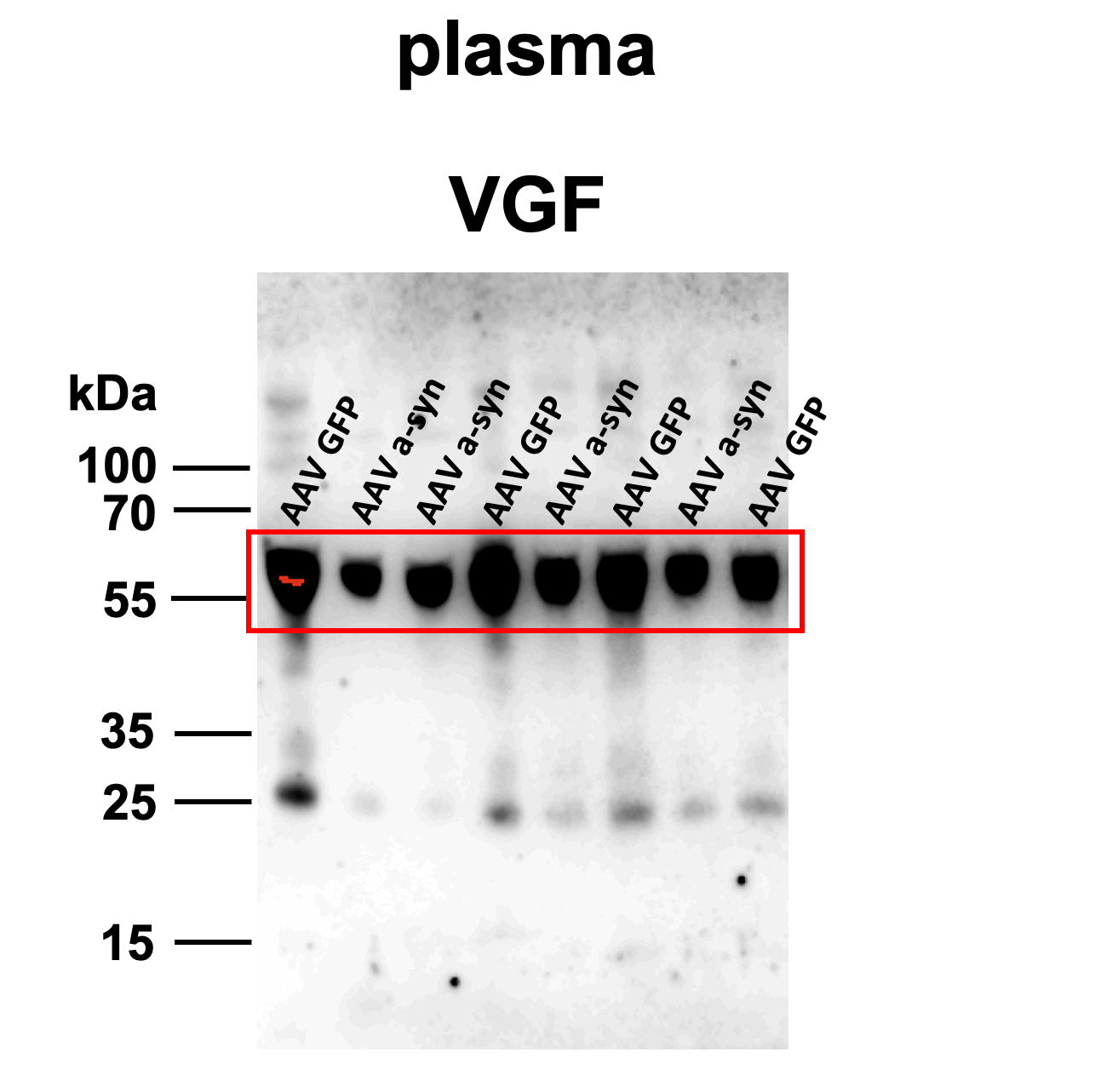

Supplement: Supplementary file 4 — Supplementary file1 (JPG 1220 KB)—Original membrane images of WB using plasma samples. Original WB membrane images of the plasma samples used to generate Fig. 6 is shown. Plasma samples were analyzed from 4 AAV-GFP-treated and 4 AAV-α-syn-treated rats. AAV-GFP: adeno-associated virus expressing green fluorescent protein; inj: injected side; con: contralateral side [file 11064_2025_4586_MOESM4_ESM.png]
